# Patterns and Determinants of Care-Seeking for Antepartum and Intrapartum Complications in Rural Bangladesh: Results from a Cohort Study

**DOI:** 10.1371/journal.pone.0167814

**Published:** 2016-12-20

**Authors:** Rasheda Khanam, Andreea A. Creanga, Alain K. Koffi, Dipak K. Mitra, Arif Mahmud, Nazma Begum, Syed Mamun Ibne Moin, Malathi Ram, Md Abdul Quaiyum, Saifuddin Ahmed, Samir K. Saha, Abdullah H. Baqui, Projahnmo Study Group in Bangladesh

**Affiliations:** 1 International Center for Maternal and Newborn Health, Department of International Health, Johns Hopkins Bloomberg School of Public Health, Baltimore, Maryland, United States of America; 2 International Centre for Diarrhoeal Disease Research, Bangladesh (ICDDR,B), Dhaka, Bangladesh; 3 Independent University, Dhaka, Bangladesh; 4 Department of Population, Family and Reproductive Health, Johns Hopkins Bloomberg School of Public Health, Baltimore, Maryland, United States of America; 5 Department of Microbiology, Dhaka Shishu Hospital, Dhaka, Bangladesh; Liverpool School of Tropical Medicine, UNITED KINGDOM

## Abstract

**Background:**

The burden of maternal complications during antepartum and intrapartum periods is high and care seeking from a trained provider is low, particularly in low middle income countries of sub-Saharan Africa and South Asia. Identification of barriers to access to trained care and development of strategies to address them will contribute to improvements in maternal health. Using data from a community-based cohort of pregnant women, this study identified the prevalence of antepartum and intrapartum complications and determinants of care-seeking for these complications in rural Bangladesh.

**Methods:**

The study was conducted in 24,274 pregnant women between June 2011 and December 2013 in rural Sylhet district of Bangladesh. Women were interviewed during pregnancy to collect data on demographic and socioeconomic characteristics; prior miscarriages, stillbirths, live births, and neonatal deaths; as well as data on their ability to make decision to go to health center alone. They were interviewed within the first 7 days of child birth to collect data on self-reported antepartum and intrapartum complications and care seeking for those complications.

Bivariate analysis was conducted to explore association between predisposing (socio-demographic), enabling (economic), perceived need, and service related factors with care-seeking for self-reported antepartum and intrapartum complications. Multivariable multinomial logistic regression was performed to examine the association of selected factors with care-seeking for self-reported antepartum and intrapartum complications adjusting for co-variates.

**Results:**

Self-reported antepartum and intrapartum complications among women were 14.8% and 20.9% respectively. Among women with any antepartum complication, 58.9% sought care and of these 46.5% received care from a trained provider. Of the women with intrapartum complications, 61.4% sought care and of them 46.5% did so from a trained provider. Care-seeking for both antepartum and intrapartum complications from a trained provider was significantly higher for women with higher household wealth status, higher literacy level of both women and their husbands, and for those living close to a health facility (<10 km). Women’s decision making ability to go to health centre alone was associated with untrained care only for antepartum complications, but was associated with both trained and untrained care for intrapartum complications.

**Conclusions:**

Nearly 40.0% of the women who experienced either an antepartum or intrapartum complications did not seek care from any provider and 11.5% -14.9% received care from untrained providers, primarily because of economic and geographic barriers to access. Development and evaluation of context specific, cost-effective, and sustainable strategies that will address these barriers to access to care for the maternal complications will enhance care seeking from trained health care providers and improve maternal health.

## Introduction

Despite substantial improvements in global maternal mortality, complications during antepartum and intrapartum periods remain high, particularly in low middle income countries (LMICs) of sub-Saharan Africa and South Asia [1–3]. The actual burden of maternal complications during antepartum and intrapartum periods is not known; the literature provides varying estimates. For every maternal death, it is estimated that about 20 women experience acute or chronic complications due to maternal causes with substantial impact on physical, psychological, social and economic outcomes [4]. According to another estimate, approximately 15.0% of all pregnant women or about 20 million women annually around the world experience acute severe obstetric complications, including haemorrhage, pre-eclampsia, eclampsia, obstructed or prolonged labour, puerperal sepsis, and abortion [2, 5, 6]. There is a need for additional studies to more precisely estimate the burden of maternal complications. In addition to affecting mothers’ own health, maternal complications have also been shown to affect the health of the fetus and newborn. The risk of perinatal mortality increases with complications such as maternal sepsis, pre-eclampsia/eclampsia, severe anaemia, placental abruption, or ruptured uterus [7, 8]. Therefore, timely recognition of antepartum and intrapartum complications and prompt care-seeking from trained providers are crucial in improving maternal as well as fetal and newborn health.

Despite World Health Organization’s recommendations that all women should receive skilled maternity care, disparities in care-seeking, particularly in LMIC are common [9]. Women’s care-seeking behaviors are influenced by a variety of individual, familial, and contextual factors. According to Anderson’s Health Behavior Model, these factors can be categorized into *predisposing*, *enabling*, related to *perceived needs* for seeking care or factors associated with health *services* [10]. Notable examples of factors shown to be associated with seeking care for maternal complications during antepartum and child birth from trained providers include availability, access and quality of health services [11, 12]. Earlier studies that examined factors associated with care seeking found that women belonging to wealthier households were more likely to seek skilled care for antepartum complications [11, 13]. Factors associated with skilled birth attendants during intrapartum period included mothers’ occupation, higher parity, complications during antepartum period, having antenatal care [14], and closer distance to a health facility [13, 15, 16]. In addition, access to skilled attendants, availability of quality care and associated medical cost remained major barriers to seeking care [11]. Improved understanding of the determinants of seeking skilled care for antepartum and intrapartum complications as well as developing strategies to improve care seeking behavior are urgently needed.

Using data from a cohort of pregnant women in rural Bangladesh followed from early pregnancy through the postpartum period, this secondary analysis examines the prevalence of key complications during antepartum and intrapartum periods and patterns and determinants of women’s care-seeking for those complications.

## Materials and Methods

### Study population

This is a secondary analysis of data from a community-based cohort study designed to identify aetiology and risk factors for community acquired infection in young infants during the first two months of life. Details of the study population and design were discussed elsewhere [17]. Briefly, the study was conducted in an established surveillance site in two rural sub-districts (Kanaighat and Zakiganj) of Sylhet division of Bangladesh between June 2011 and December 2013 in a population of about 400,000 with an approximate annual births of 10,000. The neonatal mortality rate of the study area was about 36 per 1,000 livebirths [18]. The study area was mapped using Geographic Information System (GIS). All households and household members have unique permanent identification number allowing longitudinal linkages. The study employed locally recruited women with at least tenth grade of education and trained them to serve as community health workers (CHWs). CHWs were trained on a package of maternal and newborn health (MNH) interventions that included counseling and education on preventive care during pregnancy and on maternal and newborn danger signs requiring referral for emergency care during antepartum, intrapartum, and the postpartum period; the intervention package is hereafter referred to as a MNH package. The MNH package was tested previously in this population using a randomized trial design and was shown to significantly reduce newborn mortality [19]. CHWs visited all consented women of child bearing age every two months to identify pregnancies, and followed all pregnant women through child birth and both newborns and mothers up to 60 days postpartum. CHWs visited pregnant women enrolled in the study twice during antepartum and ten times during the postpartum period to provide the MNH intervention package and to collect study related data.

### Data

During the first antepartum visit following pregnancy identification, CHWs obtained consent from women to participate in the study and collected data on women’s demographic and socioeconomic characteristics; last menstrual period; birth history including prior miscarriages/abortions, stillbirths, live births, and neonatal deaths; as well as data on their ability to make decision to go to health center alone. At the first postpartum visit, within 7 days of delivery, women were asked whether they experienced antepartum and/or intrapartum complications. There were five antepartum and six postpartum complications that were examined in this study. Specifically, women were asked if they had experienced any of the following serious complications during antepartum period: i) Any vaginal bleeding but not spotting that made their clothes wet as a sign of antepartum haemorrhage (APH); ii) fever or iii) foul smelling vaginal discharge as symptoms of infection; iv) convulsion or v) swelling of feet or face as symptoms of hypertensive disorders of pregnancy. Also, women were asked if they experienced intrapartum complications in the form of i) excessive bleeding during intrapartum period defined as bleeding that made the woman afraid of dying; ii) prolonged labour defined as labour lasting longer than 12 hours; iii) premature rupture of membranes defined as rupture of the membrane more than one hour before start of labour; iv) abnormal presentation of baby; v) convulsion; and vi) retained placenta defined as failure to deliver the placenta for more than half an hour after the delivery of the baby. The complications were the serious symptoms that the CHWs taught to the women. Women who reported one or more antepartum and/or intrapartum complications were asked if they had sought care for those complications from a health provider, either from a trained or an untrained providers. Thus, the average recall period for information for antepartum complications and related care seeking was about 5 months and for intrapartum complications was less than a week.

### Measurements

We created a household wealth index using Principal Component Analysis (PCA) method that used data on type of housing and household possessions, a methodology generally used in Demographic and Health Surveys [20]. Antenatal care (ANC) was defined as care obtained during pregnancy from a medically trained provider (Qualified doctors, Nurse, Midwife and Paramedic). Information on type of care seeking for antepartum and intrapartum complications was classified into three categories using a hierarchical algorithm: 1) trained care, if care was obtained from a medically-trained provider, i.e. a legally authorized provider such as a doctor or a nurse or a midwife or a paramedic working either at government or in private health facilities (hospitals or health centers or private clinic/chambers); 2) untrained care, if care was obtained from a non-medically trained provider, i,e. someone who was not legally authorized or regulated to provide care for maternal complications such as community health workers, traditional birth attendants, village doctors, and spiritual healers; and 3) no care. If a woman had sought care from both trained and untrained providers for the same complication, the woman was classified as having received trained care. Those who had reported antepartum and/or intrapartum complications but did not report seeking care from either trained or untrained providers were categorized as having received “no care.”

### Analysis

**Fig 1:**
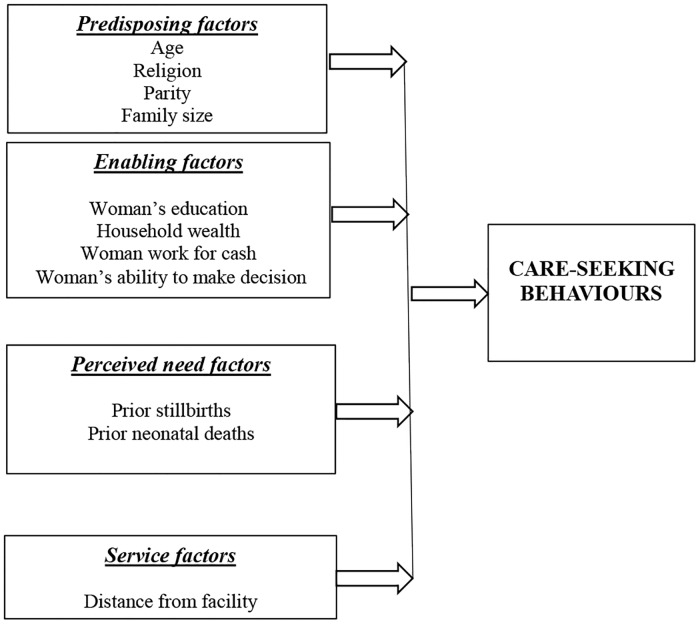
Conceptual framework. Note: Adapted from Andersen RM. Revisiting the behavioral Model and access to Medical Care: Does It Matter? Journal of Health and Social Behaviour 1995, vol. 36 (March): 1–10.

We examined the prevalence of complications during antepartum [antepartum haemorrhage (APH), high fever, foul smelling vaginal discharge and convulsions and/or swelling of the feet or face] and intrapartum period [intrapartum haemorrhage (IPH), prolonged labour, premature rupture of membrane, abnormal presentation, convulsions, and retained placenta] as well as care-seeking for reported antepartum and intrapartum complications. We adapted Andersen’s conceptual model to outline relevant factors that might have influenced care-seeking behaviors of women who experienced antepartum and/or intrapartum complications (Fig 1). *Predisposing factors* considered were women’s age, religion, parity, and family size; *enabling factors* were household wealth, women’s and their partners’ education, women’s employment and their ability to make decisions; *perceived need factors* included women’s previous obstetric history for both antepartum and intrapartum complications and experience of antepartum complications, and receipt of trained ANC care for intrapartum complications; and, *service factor* in this analysis was distance to the closest health facility that is equipped to provide basic emergency obstetric and newborn care (BEmONC). We examined unadjusted associations between the key variables of interest and care-seeking (trained, untrained, none) behaviours using Pearson’s chi-square test for independence, separately for antepartum and intrapartum complications. A p-value <0.05 was considered statistically significant. Finally, two separate multinomial logistic regression models were fitted to estimate relative risk ratios (RRR) and 95% confidence intervals (CI) for seeking trained and untrained care, respectively, compared to no care for antepartum and intrapartum complications, adjusting for factors associated significantly and at p<0.20 level in bivariate analyses. We have excluded ‘parity’ from the multinomial logistic regression models as it was highly correlated with mothers’ age. Data analysis was performed using STATA 14 (Stata Corporation 2015, College Station, TX, USA).

Approval for ethical conduct of human subject research was obtained from the Ethical Review Committee of the International Centre for Diarrhoeal Disease Research, Bangladesh (icddr,b) and the Institutional Review Board of the Johns Hopkins Bloomberg School of Public Health, USA. The present analysis uses data from a community based study known as Aetiology of Neonatal Infection in South Asia (ANISA), which had four activities. Obtaining consent was different for each activity. The activities were as follows: 1) Community-based active surveillance of pregnancies, births, and young infants (< 2 months of age); 2) Specimen collection and testing in infants with suspected serious infection; 3) Facility-based passive surveillance for serious infections in young infants; and 4) Screening a sub-cohort of healthy young infants for bacterial and viral pathogens. The present analysis was a part of activity 1 for which informed verbal consent was obtained from all participants.

CHWs obtained consent from women in their homes after confirming that women were eligible for study participation. The consent form was read aloud because many women in the study area were illiterate. After explaining the study procedures, the interviewer asked the respondent if she had any questions. If the respondent had any questions, the interviewer answered the questions and ensured that the participant understood the study procedures. The informed consent forms described that participation was voluntary and could be terminated at any time without reason and without any penalty. Verbal consent was obtained for this part of the study because it involved minimal or no risk, and waiver from a written consent did not adversely affect the rights or welfare of the study participants. The ethics committees of icddr,b in Bangladesh and Johns Hopkins Bloomberg School of Public Health University, USA had reviewed the consent form and provided approval on the consent procedure. Participants’ consent was documented in a printed copy of the consent form, which were saved in locked file cabinets.

## Results

**Fig 2:**
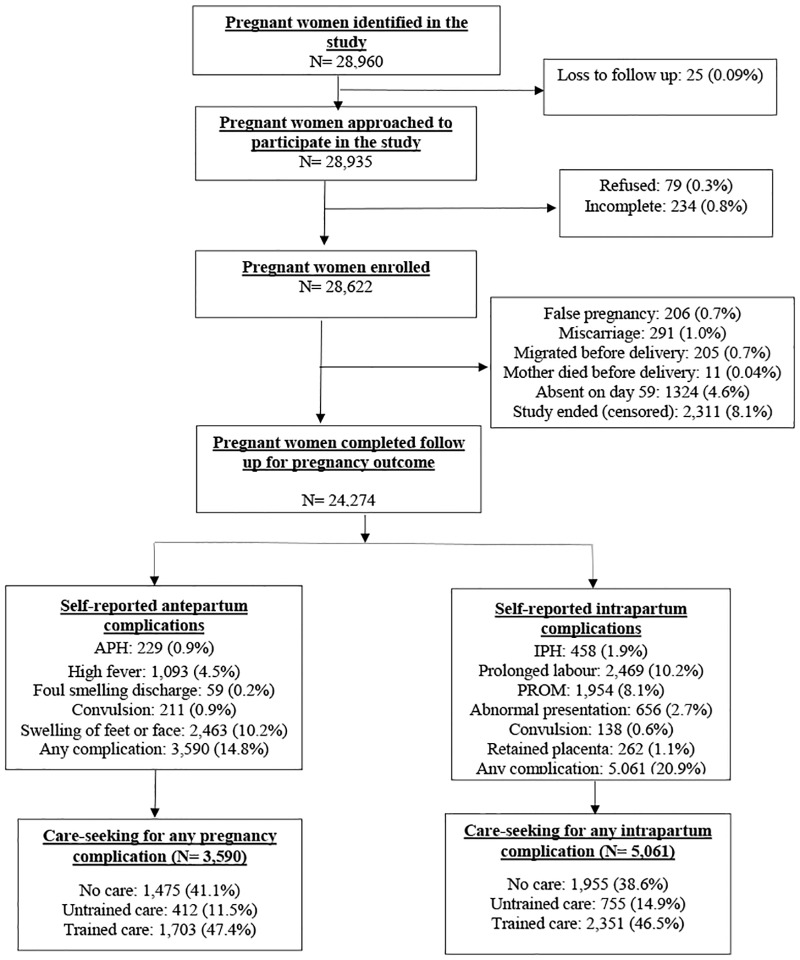
Analytic cohort of pregnant women, maternal complications and care seeking for complications. Note: APH: antepartum hemorrhage; IPH: intrapartum hemorrhage; PROM: premature rupture of membrane. Untrained care: care from community health worker; traditional birth attendant; village doctor; homeopath; or spiritual healers; trained care: care from doctor or a nurse or a midwife or a paramedic.

Of the 28,960 women who were approached to participate in the study, 79 (<0.3%) refused participation. Among 28,622 women, about 15.2% were lost to follow up. The most important reasons were censoring of women (8.1%) i.e., the woman’s pregnancy did not end when the study follow-up ended and 4.6% women were absent during scheduled visit at day 59 after delivery (Fig 2). The mean (± SD) age of enrolled women was 26.9 (± 6.0) with a range of 14–55 years. About a quarter of the study women and more than third of their husbands had no formal education.

Of the 24,274 pregnant women who completed follow up visits, about 14.8% reported at least one of the five antepartum complications. The most common antepartum complications reported were swelling of feet or face (10.2%) and high fever (4.5%) (Fig 2). About one in every five women (20.9%) had experienced any intrapartum complications (Fig 2). Prolonged labour (10.9%), premature rupture of membranes (8.1%) and abnormal presentation (2.7%) were the top three intrapartum complications reported (Fig 2). Among the 3,590 women with any of the five antepartum complications, 2,115 (58.9%) sought care; 47.4% received from a trained provider. Similarly, the majority of the women (61.4%) sought care for intrapartum complications, and nearly half (46.5%) received care from a trained provider (Fig 2). However, 41.1% and 38.6% women did not seek any care despite having had antepartum or intrapartum complications, respectively.

In bivariate analyses, care-seeking from a trained provider for antepartum complications, was significantly different by women’s age, (p < 0.05), parity (p <0.01), and household wealth status (p<0.001). Education of either women themselves or their husbands’ was also significantly associated with seeking trained care (p<0.001). Care-seeking from a trained provider was higher among women who were living within 10 km from a health facility (p <0.001) compared to women who were living ≥10 km away from a health facility Table 1.

**Table 1:** Care seeking behaviour for self-reported antepartum complications by selected characteristics of women.

Characteristics	No care	Untrained care	Trained care	p value^2^
	n = 1,475 (%)^1^	n = 412 (%)	n = 1,703 (%)	
***Predisposing factors***				
Age				
<20 years	140 (37.4)	42 (11.2)	192 (51.3)	<0.05
20–29 years	810 (40.7)	208 (10.5)	973 (48.9)	
= >30 years	525 (42.9)	162 (13.2)	538 (43.9)	
Religion				
Islam	1,423 (41.1)	402 (11.6)	1,635 (47.3)	0.30
Others	52 (40.0)	10 (7.7)	68 (52.3)	
Parity				
0	499 (39.1)	131 (10.3)	646 (50.6)	<0.01
1–2	501 (41.5)	130 (10.8)	576 (47.7)	
3–4	290 (42.8)	84 (12.4)	303 (44.8)	
= >5	185 (43.0)	67 (15.6)	178 (41.4)	
Family size				
1–4	430 (43.2)	106 (10.7)	459 (46.1)	0.41
5–6	427 (41.8)	119 (11.7)	475 (46.5)	
7–8	287 (40.6)	87 (12.3)	333 (47.1)	
≥ 9	331 (38.2)	100 (11.5)	436 (50.3)	
***Enabling factors***				
Household wealth quintile				
Lowest quintile (Poorest)	326 (51.4)	95 (15.0)	213 (33.6)	<0.001
Second lowest quintile	307 (44.1)	78 (11.2)	311 (44.7)	
Middle quintile	310 (41.9)	92 (12.4)	338 (45.7)	
Second highest quintile	268 (35.9)	96 (12.9)	383 (51.3)	
Highest quintile (Richest)	264 (34.2)	51 (6.6)	458 (59.3)	
Education				
No education	421(49.9)	122 (14.4)	301 (35.7)	<0.001
1–5 years (Primary)	537 (42.7)	163 (13.0)	557 (44.3)	
= >6 years (Secondary and above)	517 (34.7)	127 (8.5)	845 (56.8)	
Husband’s education				
No education	559 (47.8)	145 (12.4)	466 (39.8)	<0.001
1–5 years (Primary)	520 (41.7)	140 (11.2)	588 (47.1)	
= >6 years (Secondary and above)	396 (33.8)	127 (10.8)	649 (55.4)	
Work for cash				
No	1,435 (41.4)	394 (11.4)	1,640 (47.3)	0.15
Yes	40 (33.1)	18 (14.9)	63 (52.1)	
Women’s ability to make decision about:				
Can go to health center alone				
No	258 (46.0)	42 (7.5)	261 (46.5)	<0.01
Yes	1,217 (40.2)	370 (12.2)	1,442 (47.6)	
***Perceived need factors***				
Past obstetric history				
Any prior stillbirths or neonatal deaths				
No	1,320 (41.1)	360 (11.2)	1,532 (47.7)	0.309
Yes	155 (41.0)	52 (13.8)	171 (45.2)	
Sought trained ANC care for current pregnancy				
No	665 (64.8)	248 (24.2)	114 (11.1)	<0.001
Yes	810 (31.6)	164 (6.4)	1,589 (62.0)	
***Service factors***				
Distance from health facility				
< 10 km	489 (37.9)	112 (8.7)	690 (53.5)	<0.001
> = 10 km	986 (42.9)	300 (13.1)	1,013 (44.1)	

^1^ Data are row percentages;

^2^ (Pearson’s) chi-square test

Similarly, care-seeking for intrapartum complications from a trained provider was significantly associated with women’s age (p<0.001), parity (p<0.001), education of both themselves and their husbands’ (p <0.001), and households wealth status (p <0.001). In addition, women who experienced any antepartum complications (p <0.001) and who received antenatal care (ANC) from a trained provider during the current antepartum were more likely to seek trained care for intrapartum complications. The proportion of women who sought trained care for intrapartum complications was significantly higher (p<0.001) among women who lived within 10 km of a health facility compared to women who were living ≥10 km away from a health facility Table 2.

**Table 2:** Care seeking behavior for self-reported intrapartum complications by selected characteristics of women.

Characteristics	No care	Untrained care	Trained care	p value^2^
	n = 1,955 (%)^1^	n = 755 (%)	n = 2,351 (%)	
***Predisposing factors***				
Age				
<20 years	227 (34.8)	104 (15.9)	322 (49.3)	<0.001
20–29 years	1,134 (37.9)	418 (14.0)	1,443 (48.2)	
= >30 years	594 (42.0)	233 (16.5)	586 (41.5)	
Religion				
Islam	1,888 (39.0)	728 (15.1)	2,220 (45.9)	<0.001
Others	67 (29.8)	27 (12.0)	131 (58.2)	
Parity				
0	658 (30.2)	325 (14.9)	1,197 (54.9)	<0.001
1–2	714 (44.7)	221 (13.8)	664 (41.5)	
3–4	399 (46.3)	134 (15.6)	329 (38.2)	
= >5	184 (43.8)	75 (17.9)	161 (38.3)	
Family size				
1–4	551 (38.7)	217 (15.2)	657 (46.1)	0.38
5–6	564 (40.2)	220 (15.7)	620 (44.2)	
7–8	379 (38.6)	137 (14.0)	466 (47.5)	
≥ 9	461 (36.9)	181 (14.5)	608 (48.6)	
***Enabling factors***				
Household wealth quintile				
Lowest quintile (Poorest)	423 (51.8)	131 (16.0)	263 (32.2)	<0.001
Second lowest quintile	434 (44.5)	169 (17.3)	372 (38.2)	
Middle quintile	412 (41.1)	158 (15.8)	433 (43.2)	
Second highest quintile	365 (34.2)	149 (14.0)	552 (51.8)	
Highest quintile (Richest)	321 (26.8)	148 (12.3)	731 (60.9)	
Education				
No education	513 (48.7)	175 (16.6)	366 (34.7)	<0.001
1–5 years (Primary)	726 (43.0)	279 (16.5)	683 (40.5)	
= >6 years (Secondary and above)	716 (30.9)	301 (13.0)	1,302 (56.1)	
Husband’s education				
No education	727 (45.9)	262 (16.5)	595 (37.6)	<0.001
1–5 years (Primary)	713 (41.0)	278 (16.0)	748 (43.0)	
= >6 years (Secondary and above)	515 (29.6)	215 (12.4)	1,008 (58.0)	
Work for cash				
No	1,905 (38.6)	739 (15.0)	2,290 (46.4)	0.76
Yes	50 (39.4)	16 (12.6)	61 (48.0)	
Women’s ability to make decision about				
Going to health center alone				
No	385 (47.0)	86 (10.5)	348 (42.5)	<0.001
Yes	1,570 (37.0)	669 (15.8)	2,003 (47.2)	
***Perceived need factors***				
Past obstetric history				
Prior stillbirths or neonatal deaths				
No	1,758 (38.2)	686 (14.9)	2,153 (46.8)	0.17
Yes	197 (42.5)	69 (14.9)	198 (42.7)	
Any antepartum complication for current antepartum				
No	1,505 (39.7)	594 (15.7)	1,697 (44.7)	<0.001
Yes	450 (35.6)	161 (12.7)	654 (51.7)	
Received trained ANC care for current antepartum				
No	740 (52.6)	265 (18.9)	401 (28.5)	<0.001
Yes	1,215 (33.2)	490 (13.4)	1,950 (53.4)	
ANC visits				
1–2	629 (39.9)	244 (15.5)	703 (44.6)	<0.001
3–4	399 (30.4)	144 (11.0)	770 (58.6)	
5+	174 (23.8)	98 (13.4)	460 (62.8)	
Missing	13 (38.2)	4 (11.8)	17 (50.0)	
***Service factors***				
Distance from health facility				
< 10 km	733 (39.0)	202 (10.7)	947 (50.3)	<0.001
> = 10 km	1,222 (38.4)	553 (17.4)	1,404 (44.2)	

^1^ Data are row percentages;

^2^ (Pearson’s) chi-square test

In multivariable multinomial regression analyses, for both antepartum and intrapartum complications, *predisposing factors*, such as age was not associated with seeking either trained or untrained care Table 3. For antepartum complications, three *enabling factors*: household wealth, education of women, and husbands’ education were significantly associated with seeking trained care Table 3. Compared to women in the lowest wealth quintile, women in the second highest and highest wealth quintiles were about two folds more likely (RR, 95% CI: 1.7, 1.30–2.16 and RR, 95% CI: 1.8, 1.40–2.39, respectively) to seek trained care. Women in the highest wealth quintile were significantly less likely to seek untrained care (RR, 95% CI: 0.6; 0.40–0.95). Women who attended school for 1 to 5 years (RR, 95% CI: 1.3, 1.03–1.55) and who attended sixth grade or higher (RR, 95% CI: 1.7, 1.34–2.13) were more likely to seek trained care. Woman with a husband of sixth grade or higher education were more likely to seek care, both trained (RR, 95% CI: 1.2, 1.01–1.54) and untrained (RR, 95% CI: 1.5, 1.11–2.12). For antepartum complications, women’s ability to make decision to go to health center alone was associated with untrained care (RR, 95% CI: 1.9, 1.36–2.76) Table 3.

**Table 3:** Multinomial logistic regression of factors associated with care-seeking from untrained and trained providers for self-reported antepartum complications.

	Antepartum complications		Intrapartum complications	
Variables	Trained vs No care	Untrained vs No care	Trained vs No care	Untrained vs No care
	Relative Risk Ratio (RRR), 95% CI	Relative Risk Ratio (RRR), 95% CI	Relative Risk Ratio (RRR), 95% CI	Relative Risk Ratio (RRR), 95% CI
***Predisposing factor***				
Age				
<20 years	1.0; 0.77–1.31	0.9; 0.63–1.43	1.0; 0.87–1.18	1.0; 0.77–1.40
20–29 years	1.0; 0.82–1.15	0.8; 0.64–1.06	1.0; 0.80–1.26	0.8; 0.68–1.03
= >30 years	Ref	Ref	Ref	Ref
***Enabling factors***				
Household wealth				
Lowest quintile (Poorest)	Ref	Ref	Ref	Ref
Second lowest quintile	1.4, 1.10–1.78	0.9; 0.61–1.22	1.2; 0.93–1.43	1.2; 0.92–1.60
Middle quintile	1.4 1.09–1.77	1.0; 0.69–1.38	1.2; 0.97–1.51	1.1; 0.84–1.50
Second highest quintile	1.7, 1.30–2.16	1.2; 0.81–1.66	1.5; 1.18–1.88	1.2; 0.85–1.57
Highest quintile (Richest)	1.8, 1.40–2.39	0.6; 0.40–0.95	1.9; 1.49–2.43	1.3; 0.93–1.80
Education				
No education	Ref	Ref	Ref	Ref
1–5 years	1.3, 1.03–1.55	1.0; 0.77–1.39	1.0; 0.83–1.21	1.1; 0.84–1.37
= >6 years	1.7; 1.34–2.13	0.8; 0.54–1.09	1.3; 1.05–1.60	1.0; 0.81–1.41
Husband’s education				
No education	Ref	Ref	Ref	Ref
1–5 years	1.1; 0.90–1.30	1.1; 0.86–1.49	1.0; 0.87–1.21	1.0; 0.83–1.26
= >6 years	1.2; 1.01–1.54	1.5; 1.10–2.12	1.4; 1.14–1.65	1.0; 0.80–1.32
Work for cash				
No	Ref	Ref	Ref	Ref
Yes	1.4; 0.93–2.13	1.7; 0.94–3.01	0.9; 0.62–1.37	0.9; 0.52–1.65
Women’s decision making about				
Going to health center alone				
No	Ref	Ref	Ref	Ref
Yes	1.1; 0.89–1.32	1.9; 1.36–2.76	1.3; 1.05–1.49	1.8; 1.4–2.28
***Perceived need factor***				
Obstetric history				
Previous stillbirths or neonatal deaths				
No	Ref	Ref	Ref	Ref
Yes	1.2; 0.93–1.55	1.2; 0.82–1.65	1.1; 0.88–1.38	1.0; 0.71–1.29
Any Antepartum complication during current antepartum				
No			Ref	Ref
Yes			1.2; 1.07–1.43	0.9; 0.72–1.09
ANC trained care for current antepartum				
No			Ref	Ref
Yes			2.2; 1.90–2.58	1.1; 0.86–1.27
***Service need factor***				
Distance from facility				
< 10 km	1.4; 1.22–1.65	0.8; 0.60–0.98	1.2; 1.05–1.37	0.6; 0.53–0.78
> = 10 km	Ref	Ref	Ref	Ref

For intrapartum complications, four *enabling factors*: household wealth, education of women, husbands’ education, women’s ability to go to health center alone were significantly associated with seeking care from a trained provider Table 3. Women belonging to the two highest wealth quintiles were more likely (RR, 95% CI: 1.5, 1.18–1.88 & RR, 95% CI: 1.9, 1.49–2.43) to seek trained care. Compared to no education, sixth grade or higher education of women (RR, 95% CI: 1.3, 1.05–1.60) and their husbands’ (RR, 95% CI: 1.4, 1.15–1.66) were more likely to be associated with seeking trained care Table 3. Women who reported having ability to go to health center alone were more likely to seek both trained (RR, 95% CI: 1.3, 1.05–1.49) and untrained care (RR, 95% CI: 1.8, 1.35–2.28) for intrapartum complications Table 3.

For intrapartum complications, women who experienced any complications during the current pregnancy (RR, 95% CI: 1.2, 1.07–1.43) and women who received trained ANC care were more likely (RR, 95% CI: 2.2, 1.90–2.58) to seek trained care. With regard to *service factor*, women who lived within 10km from a health facility were more likely to receive trained care for both antepartum (RR, 95% CI: 1.4, 1.22–1.65) and intrapartum complications (RR, 95% CI 1.2, 1.05–1.37) compared to women who were living ≥10 km away from a health facility Table 3.

## Discussion

In this population-based cohort of Bangladeshi women, the burden of self-reported serious antepartum and intrapartum complications was high at 14.8% and 20.9%, respectively. Although a majority of women sought care and almost half did so from a trained provider for both antepartum and intrapartum complications. For antepartum and postpartum complications, 41.1% and 38.6% of women did not seek any care, respectively.

We adopted the Andersen’s behavioural model to examine the contribution of different types of factors that contributed or hindered seeking trained care for antepartum and intrapartum complications. The model postulates predisposing, enabling, perceived need and service related factors that might affect care-seeking behaviors of women. The present study documented that enabling and service related factors were important determinants for use of trained care for antepartum and intrapartum complications. This finding is important because enabling and service related factors are more modifiable than predisposing and perceived needs factors (Andersen 1995).

The enabling and service related factors, higher household wealth status, higher literacy level of both women and their husbands, and living close to a health facility (<10 km) were positively associated with seeking trained care for both antepartum and intrapartum complications. Women’s decision making ability to go to health centre alone was associated with untrained care only for antepartum complications, but was associated with both trained and untrained care for intrapartum complications.

Care-seeking from a trained provider, both for antepartum (47.4%) and intrapartum (46.5%) complications, in our study was higher compared to a similar study conducted among women in rural Gaibandha and Rangpur districts in Bangladesh between 2007 and 2011, where only a quarter of the women sought trained care [15]. Also, the care-seeking rate in our study was higher compared to findings of a national survey, conducted 10 years earlier in Bangladesh, where one in three women reported receiving care from a qualified provider for their life threatening antepartum complications [11]. However, the care-seeking rate from a trained provider for obstetric complications that we observed was similar to studies conducted in Bangladesh and Ethiopia [13, 21]. The higher use of trained care in our study population compared to other Bangladesh studies might partly be due to time lag between the studies and partly be the impact of a MNH intervention package that we have been implementing in this population for more than a decade. The intervention package involves home visits by CHWs and counseling pregnant women on antepartum and intrapartum complications and care seeking from trained providers [19].

This analysis revealed that improved socio-economic status appeared to enable care-seeking from trained providers for antepartum and intrapartum complications. Many studies in Bangladesh and elsewhere have shown socio-economic disparities in care seeking [22] with some studies documenting positive association between improved wealth status and seeking trained maternity care [9, 11, 12, 15]. In our study, women in the highest two wealth quintiles were about two times more likely to seek trained care both for reported antepartum and intrapartum complications [15], which was in agreement with findings of earlier studies conducted in Bangladesh and in other countries [13, 15].

Care-seeking from a trained provider was associated with both women’s and husband’s literacy for both antepartum and intrapartum complications; these findings were consistent with the findings of an earlier study in Bangladesh where women’s literacy was associated with seeking care from a qualified provider for obstetric complications [15]. Women’s literacy and household wealth status are important factors and are considered as proxies of women’s decision making ability, empowerment and affordability for maternal health care utilization [23, 24]. In our study, women who were living in a distance of less than 10 km of a health facility were more likely to seek trained care for both antepartum and intrapartum complications. This finding is consistent with findings of a number of studies conducted in Bangladesh and elsewhere [12, 15]. In a study conducted between 2007 and 2011 in Bangladesh, Sikder et al showed that living within 10 km from the health facility was associated with seeking formal care for intrapartum complications [15]. Another cross-sectional study conducted in Tanzania documented a significant association between shorter distance to a health facility and accessing skilled assistance for intrapartum care [12]. Lack of access to transport and associated cost along with out of pocket expenditure in the health facility remain persistent challenges in many developing countries [11, 13].

Women with antepartum complications and those having received ANC from a trained provider for the index pregnancy were more likely to seek trained care for intrapartum complications [13, 25] than their counterparts. This association may be due to residual confounding from unmeasured heterogeneity between women who sought ANC and who did not. For example, women who sought ANC and trained care for intrapartum complications might have higher awareness, ability to seek care, or might have higher perceived needs. Our analysis is adjusted for some of these variables related to care-seeking, however, there are many more variables that we could not measure in this study.

The main limitation of our study is that the analyses are based on self-reported antepartum and intrapartum complications which tend to misestimate the prevalence of complications due to misclassification [26, 27]. However, we attempted to use potentially serious and easily recognizable and reportable antepartum and intrapartum complications by women themselves. In developing country settings where utilization of ANC and delivery care at health facility is low, clinical diagnosis of maternal complications remains a major challenge. The study could not include about 15.2% women of which 8.1% were censored, 4.6% were absent on day 59 and the rest could not complete interview for other reasons (Fig 2). This might limit the generalizability of the study to an extent. However, we do not consider that the exclusion of these women created any selectivity bias; the women who were lost to follow up were not different from the women who completed the study in terms of background characteristics (results not shown). The strengths of the study include large sample size, population-based prospective surveillance with independent identification of pregnancies and short recall period that might have minimized recall errors of reported complications and care-seeking behaviours.

The key programmatic findings from this study is that care-seeking from trained providers for antepartum and intrapartum complications is associated with access to care, both geographic and financial. World Health Organization member countries embraced the concept of universal coverage for maternity care by 2005, however, many LMICs have yet to achieve this goal [28]. This is mainly due to numerous barriers that hamper access to needed health services for pregnant and postpartum women. Results from a verbal and social autopsy study conducted in the same population where the present study was conducted revealed cost as one of the main barriers for seeking trained care for neonatal illness [29]. Interventions, such as demand side financing including voucher schemes or conditional cash transfer, have shown promise in improving maternal health care utilization by improving access and reducing inequity in low and middle income countries [22, 30–34]. However, these interventions need to be tailored to local contexts for effectiveness and sustainability. In addition, these interventions require an effective primary health care systems to be in place [31, 32]. Although we could not study, poor quality of care is also a major barrier to care-seeking and an important impediment to improving maternal health [25, 35, 36].

Identifying factors that facilitate care-seeking from trained provider for maternal complications is crucial [37]. We recommend that programs develop and evaluate context specific, cost-effective, and sustainable strategies to improve access to high quality care from trained health care providers for women with antepartum and intrapartum complications.
